# Alterations of Bioactive Lipid Profiles in the Retina Following Traumatic Optic Neuropathy in Mice

**DOI:** 10.3390/biom15101450

**Published:** 2025-10-14

**Authors:** Min Young Kim, Nandini Koneru, Gieth Alahdab, Michael Risner, Ahmed S. Ibrahim, Krishna Rao Maddipati, Mohamed Al-Shabrawey

**Affiliations:** 1 Eye Research Center and Foundational Medical Studies, Oakland University William Beaumont School of Medicine (OUWB-SOM), Rochester, MI 48309, USA; minyoungkim@oakland.edu (M.Y.K.); nkoneru@oakland.edu (N.K.); giethalahdab@oakland.edu (G.A.); mlrisner@oakland.edu (M.R.); 2 Eye Research Institute, Oakland University, Rochester, MI 48309, USA; 3 Department of Ophthalmology, Visual, and Anatomical Sciences, School of Medicine, Wayne State University, Detroit, MI 48201, USA; ahmed.ibrahim@wayne.edu; 4 Department of Biochemistry, Faculty of Pharmacy, Mansoura University, Mansoura 35516, Egypt; 5 Department of Pharmacology, School of Medicine, Wayne State University, Detroit, MI 48201, USA; 6 Bioactive Lipids Research Program, Department of Pathology, Wayne State University School of Medicine, Detroit, MI 48202, USA; aj2642@wayne.edu; 7 Lipidomics Core Facility, Wayne State University School of Medicine, Detroit, MI 48202, USA

**Keywords:** traumatic optic neuropathy, lipoxygenase, hydroxyeicosatetraenoic acid, 12/15-HETEs, 12/15-LOX, controlled orbital impact, bioactive lipids, arachidonic acid

## Abstract

Traumatic optic neuropathy (TON) causes vision loss through compression and contusion, yet there is no consensus on the most effective treatment. Polyunsaturated fatty acid (PUFA)-derived bioactive lipids metabolized by lipoxygenase (LOX), cytochrome P450 (CYP), and cyclooxygenase (COX) enzymes are known mediators of inflammation and neurodegeneration. However, their role in TON-related retinal pathology remains unclear. Controlled orbital impact (COI) was used to induce unilateral TON in mice with controlled velocity (2–3 m/s), with the fellow eye serving as an internal control. Retina tissues were collected three days post-injury and analyzed by LC/MS to quantify bioactive lipid metabolites from ω−6 and ω−3 PUFAs. Statistical analysis was performed using paired, nonparametric Wilcoxon signed-rank tests with Benjamini–Hochberg false discovery rate (FDR) correction. Results showed that among 38 reliably detected metabolites, no individual lipid showed a statistically significant difference between TON and control eyes after FDR correction (q < 0.05). However, both individual and pathway-level analysis revealed consistent trends toward increased expression of LOX- and CYP-derived metabolites across FDA PUFA substrates, including arachidonic acid (AA), linoleic acid (LA), and docosahexaenoic acid (DHA). These findings support further investigation into lipid-mediated inflammation in TON and its potential as a therapeutic target, particularly through expanding both the sample size and the post-TON time periods.

## 1. Introduction

Traumatic optic neuropathy (TON) is a vision-threatening condition caused by damage to the optic nerve, leading to the degeneration of retinal ganglion cells (RGC) and their axons [1,2,3,4,5]. It is a leading cause of vision loss among civilian and military populations following head or ocular trauma forces, yet no effective therapies currently exist to halt progression. Currently, TON is treated by surgery and corticosteroid therapy, which are often not effective or involve risks associated with the intervention. Surgical decompression, though sometimes indicated, poses risks such as bone loss and infection [6]. The less invasive endoscopic transnasal/transseptal optic canal decompression shows promise of fewer complications but requires highly experienced surgeons to attain positive visual acuity outcomes [7]. High-dose corticosteroids have been commonly used as a first-line therapy, but have failed to show benefit in randomized trials and may even reduce RGC axon survival [8,9,10,11]. With a 0–48% rate of spontaneous improvement in case literature reports, many ophthalmologists and patients opt for conservative management with no acute treatment [12]. This underscores an urgent need to identify novel therapeutic targets to treat TON.

To identify therapeutic targets, we need to understand the mechanisms piqued by TON. Out previous work using a controlled orbital impact (COI) model demonstrated functional deficits in the scotopic threshold response of the electroretinogram (ERG), implicating dysfunction of proximal retinal neurons, including RGCs [13,14]. This was supported by a reduction in the RGC marker brain-specific homeobox/POU domain protein 3A (BRN3A) and enhanced the expression of the immune response protein, ionized calcium-binding adapter molecule 1 (Iba1), which is a marker of microglia and macrophages [13]. These findings suggest that both neuronal injury and an inflammatory response from TON may contribute to RGC loss.

Inflammation is believed to play a central role in TON pathogenesis, as reflected by the clinical use of corticosteroids [15]. In both human and experimental traumatic brain injury (TBI), trauma induces pro-inflammatory responses via aberrant metabolism of polyunsaturated fatty acids (PUFAs) [16,17,18,19,20]. Because TBI may also result in vision loss through optic nerve damage, similar inflammatory lipid pathways may be relevant in TON. This idea is justified by our previously published data where we found that oxygen-induced retinal ischemia enhances the catabolism of ω−3 and ω−6 PUFAs, specifically arachidonic acid (C20:4n−6, AA), by 12/15-lipoxygenases (LOX), producing pro-inflammatory metabolites, including 12- and 15-hydroxyeicosatetraenoic acids (12/15-HETEs) [21]. Moreover, recent data from our laboratory indicates that 12/15-HETEs contribute to the activation of retinal Müller cells, which is often an indicator of inflammation [22].

Despite this mechanistic rationale, the role of PUFA-derived inflammatory and neuroprotective bioactive lipids in TON remains largely unexplored. In this study, we performed targeted lipidomic analysis to profile dysregulations in retinal PUFA metabolites following TON, focusing on products of the LOX, cytochrome P450 (CYP), and cyclooxygenase (COX) enzymatic pathways. Using an established mouse model of indirect TON [13], we aimed to assess whether bioactive lipid dysregulation could reveal candidate pathways involved in TON-related retinal inflammation.

## 2. Materials and Methods

In order to study the effect of TON on bioactive lipid levels of the retina, the controlled orbital impact (COI) model previously developed in our lab was used to induce TON in mice [13]. This model controls for variabilities and other limitations that exist in other models of TON, such as optic nerve crush, optic nerve axotomy, blast injury, and sonication-induced TON [13,23,24,25,26]. After inducing TON, mouse retinas were collected for lipidomic analysis to understand various lipid metabolic enzyme activities.

### 2.1. Animals

All procedures were approved by the Institutional Animal Care and Use Committee of Augusta University and were in accordance with the Association for Research in Vision and Ophthalmology (ARVO) Statement for the Use of Animals in Ophthalmic and Vision Research. We used 8- to 10-week-old male C57BL/6J (#000664, The Jackson Laboratory, Bar Harbor, ME, USA) mice, which were housed in a temperature- and humidity-controlled room under a 12:12-h light-dark cycle and provided water and food as desired.

### 2.2. Modeling TON in Mice

To model TON in anesthetized (2% isoflurane) mice, we modified a controlled impact device (PinPoint PCI3000 Precision Cortical Impactor; Hatteras Instruments, Cary, NC, USA) as previously described [13]. We restrained mice using a stereotaxic apparatus equipped with ear and bite bars, exposed the optic nerve by retracting the extraocular tissues, and directed the impactor to the nerve located 2–3 mm from the posterior pole of the globe. We delivered a single impact to the optic nerve of the right eye and the contralateral eye served as a control. The impact duration was 100 ms, the impact velocity ranged from 2.0- to 3.0-m/s, and contusion depth was 0.6 mm [13].

### 2.3. Assessment of PUFA Metabolites by Liquid Chromatography/Mass Spectrometry (LC/MS)

Three days after delivering injury to the optic nerve, retinas were harvested and immediately snap frozen in liquid nitrogen to quench enzymatic activity rapidly and preserve lipid composition. Samples were stored at −80 °C and shipped on dry ice to the Wayne State University Lipidomic Core Facility for targeted lipidomic analysis via LC/MS.

Tissues were homogenized in phosphate buffer (pH 7.2) at a 1:9 (*w*/*v*) ratio using a bead-based homogenizer (Precellys, Bertin Technologies). Homogenates were spiked with 5 ng each of 15(S)-HETE-d_8_, leukotriene B4-d_4_, resolvin D2-d_5_, 14(15)-EpETrE-d_11_, and prostaglandin E1-d_4_ as internal standards. Lipids were extracted using solid-phase extraction (SPE) with C18 cartridges as previously described [27,28], eluted with methanol, dried under nitrogen, and reconstituted in 50 μL of methanol/25 mM ammonium acetate (1:1, *v*/*v*) for analysis. Chromatographic separation was performed on a Shimadzu Prominence XR system using a Luna C18 column (3 µm, 2.1 × 150 mm; Shimadzu, Somerset, NJ, USA). A gradient of methanol–water–acetonitrile (A: 10:85:5; B: 90:5:5, both with 0.1% ammonium acetate) was applied at 0.2 mL/min over 17 min.

Mass spectrometry was performed on a SCIEX QTRAP 5500 (AB SCIEX, Singapore) operating in negative electrospray ionization mode with optimized instrument parameters. Lipid mediators were detected by scheduled multiple reaction monitoring (MRM) using 125 transitions for 156 lipid species. Each transition was monitored within a 120-s retention window, with optimized collision energies and cell exit potential. Identification of analytes was confirmed by retention time matching with authentic standards and enhanced product ion (EPI) spectra.

Data were acquired with Analyst 1.6.2 software and quantified using MultiQuant 3.0.3 (AB SCIEX, Singapore). Peak areas were normalized to internal standard signals for recovery and injection variability, and final analyte levels were normalized to total protein content. This targeted lipidomics panel focused on LOX, CYP, and COX-derived metabolites of AA, linoleic acid (C18:2n−6, LA), docosahexaenoic acid (C22:6n−3, DHA), and eicosapentaenoic acid (C20:5n−3, EPA). The full list of MRM transitions, including retention times, precursor/product ion pairs, and internal standards, is provided in Supplementary Table S1.

### 2.4. Statistical Analysis

To assess changes in lipid metabolites expression following TON, fold changes (FC) were calculated for each individual metabolite by dividing the value in the TON-affected retina by the value in the paired control retina from the same mouse (TON/control). This within-animal comparison preserved biological pairing and minimized inter-mouse variability. Statistical significance was determined using paired, two-tailed Wilcoxon signed-rank tests. False discovery rate (FDR) correction was applied using the Benjamini–Hochberg procedure to account for multiple testing across all detected individual metabolites, with significance defined as q < 0.05.

For pathway-level analyses, metabolites were grouped a priori by enzymatic origin (e.g., LOX, CYP, COX) based on established biosynthetic pathways. For each mouse, the average log_2_FC across all metabolites within each pathway was calculated to represent pathway-level activity. These per-mouse pathway averages were then tested against zero using paired Wilcoxon signed-rank tests. Given the exploration nature of this secondary analysis and the smaller number of comparisons, FDR correction was applied with a more permissive threshold of q < 0.10 to identify candidate pathways of interest. All statistical analyses were performed using GraphPad Prism Version 10.5.0 software (Graphpad Software Inc., La Jolla, CA, USA).

## 3. Results

**Figure 1:**
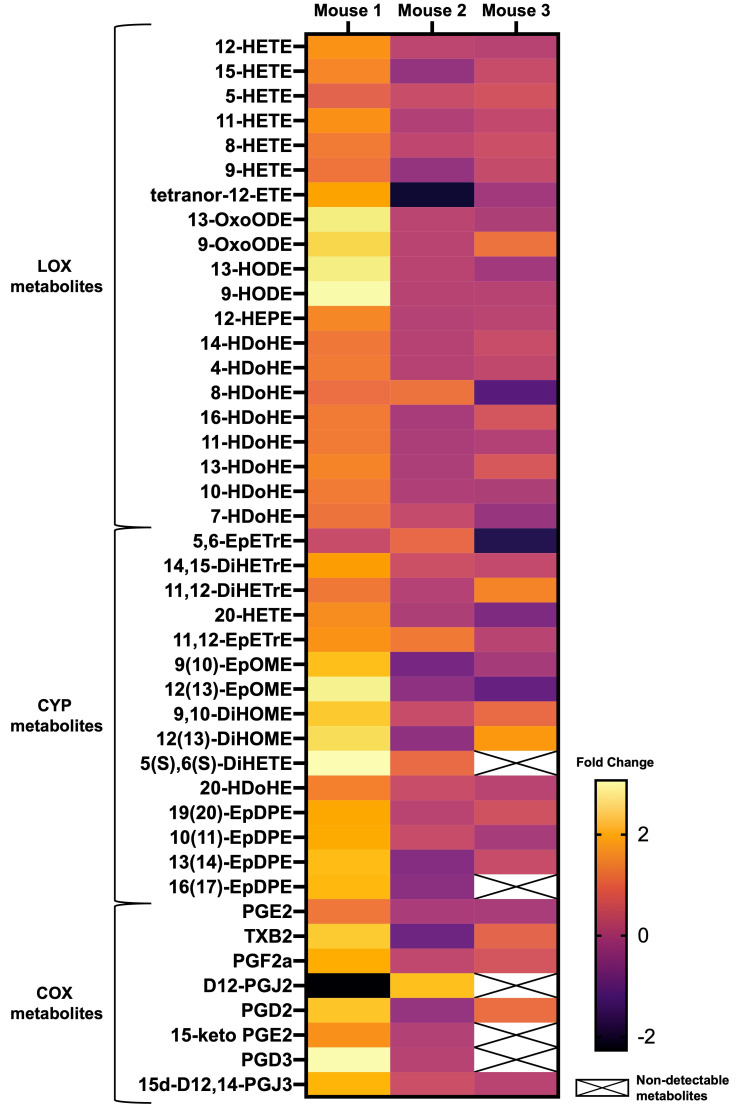
Heatmap of bioactive lipid mediator expression in TON-affected eyes relative to contralateral control eyes (*n* = 3 mice). Log_2_ fold changes (TON/Control) are shown for individual metabolites across three biological replicates. Metabolites are grouped by enzymatic pathways: lipoxygenase (LOX), cytochrome P450 (CYP), and cyclooxygenase (COX). Missing data (non-detectable metabolites) are indicated with cross-hatched boxes. Color scale represents log_2_ fold change from −2 (downregulated) to +2 (upregulated) with log_2_ values > 0 indicating increased expression.

Fold changes in retinal bioactive lipid expression following TON are visualized as a heat map in Figure 1. Based on known metabolic pathways (Figure S1), detected lipids were categorized by their metabolizing enzymes: LOX, CYP, and COX. Of the 43 bioactive lipid metabolites detected by LC/MS, five were excluded from individual metabolite-level statistical analysis due to missing values. Among the 38 metabolites included in the analysis, none showed a statistically significant difference in expression between TON and control eyes after FDR correction (q < 0.05).

**Figure 2:**
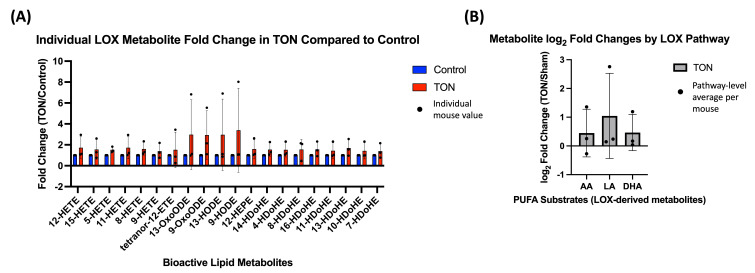
Lipoxygenase (LOX)-derived bioactive lipid changes in TON retinas. (**A**) Individual metabolite fold changes (TON vs. control) for bioactive lipids metabolized via the LOX enzymatic pathway. Bars represent the mean fold change (TON/Sham) for each metabolite (*n* = 3 mice), with error bars showing standard deviation and dots representing individual mouse fold change values. Fold changes were calculated using raw expression values, and values were statistically compared to 1 using paired Wilcoxon signed-rank tests. No individual metabolites reached statistical significance after FDR correction (q < 0.05). (**B**) Pathway-level analysis of LOX-derived metabolites grouped by their polyunsaturated fatty acid (PUFA) substrates—arachidonic acid (AA), linoleic acid (LA), and docosahexaenoic acid (DHA). Bars show the mean log_2_ fold change (TON/Sham) within each PUFA group. Each dot represents a single mouse, calculated as the average log_2_ fold change across all LOX-derived metabolites from that PUFA in that mouse. Log_2_ values > 0 indicate increased expression in TON eyes. While all PUFA groups showed a trend toward increased LOX activity, no group reached statistical significance (q < 0.10).

To further explore potential pathway-specific changes, metabolites were grouped based on their precursor PUFAs, including ω−6 (AA and LA) and ω−3 (EPA and DHA). This allowed for more targeted evaluation of specific enzymatic activity within each PUFA-enzyme axis. For example, LOX-derived metabolites were first assessed individually (Figure 2A) and then grouped by their PUFA substrates to evaluate pathway-level log_2_ FCs (Figure 2B). While no comparison reached statistical significance, both the individual LOX-derived metabolites (compared to a null value of 1) and the PUFA-grouped LOX pathway averages (compared to 0 after log_2_ transformation) showed consistent trends toward increased expression in TON eyes.

**Figure 3:**
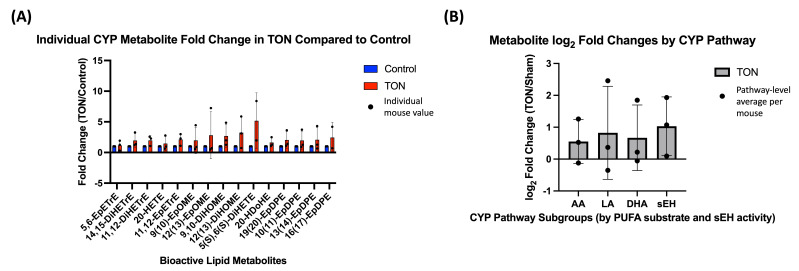
Cytochrome P450 (CYP)-derived bioactive lipid changes in TON retinas. (**A**) Individual metabolite fold changes (TON vs. control) for bioactive lipids metabolized via the CYP enzymatic pathway. Bars represent the mean fold change (TON/Sham) for each metabolite (*n* = 3 mice), with error bars showing standard deviation and dots representing individual mouse fold change values. Fold changes were calculated using raw expression values, and values were statistically compared to 1 using paired Wilcoxon signed-rank tests. No individual metabolites reached statistical significance after FDR correction (q < 0.05). (**B**) Pathway-level analysis of CYP-derived metabolites grouped by PUFA substrate—arachidonic acid (AA), linoleic acid (LA), and docosahexaenoic acid (DHA)—as well as a subgroup of metabolites further processed by soluble epoxide hydrolase (sEH), an enzyme downstream of CYP activity. Bars represent the mean log_2_ fold change (TON/Sham) for each subgroup. Each dot represents a single mouse, calculated as the average log_2_ fold change across all relevant metabolites. Log_2_ values > 0 indicate increased expression in TON eyes. While all subgroups trended upward, none reached statistical significance (q < 0.10).

CYP-derived metabolites were similarly analyzed both individually and by PUFA substrate grouping. Individual fold changes for all detected CYP metabolites are shown in Figure 3A, and pathway-level log_2_ FC grouped by PUFA origin (AA, LA, DHA) are shown in Figure 3B. In addition, a subgroup of downstream metabolites generated via soluble epoxide hydrolase (sEH), an enzyme that acts on CYP-derived epoxides, was analyzed separately to evaluate secondary processing within the CYP pathway.

Although none of the comparisons reached statistical significance after FDR correction, the majority of individual CYP metabolites showed FC >1 in TON eyes, and pathway-level averages for all PUFA subgroups, including sEH products, exhibited positive log_2_ FC.

**Figure 4:**
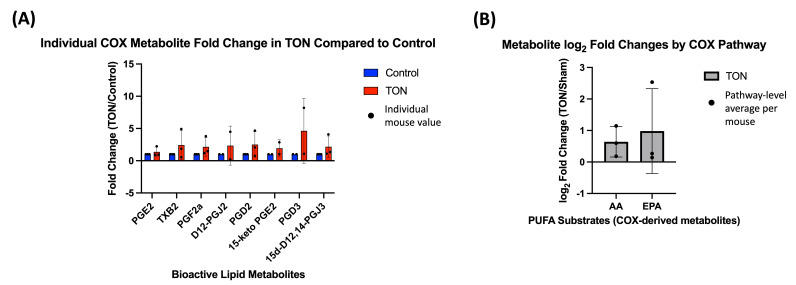
Cyclooxygenase (COX)-derived bioactive lipid changes in TON retinas. (**A**) Individual metabolite fold changes (TON vs. control) for bioactive lipids metabolized via the COX enzymatic pathway. Bars represent the mean fold change (TON/Sham) for each metabolite (*n* = 3 mice), with error bars showing standard deviation and dots representing individual mouse fold change values. Fold changes were calculated using raw expression values, and values were statistically compared to 1 using paired Wilcoxon signed-rank tests. No individual metabolites reached statistical significance after FDR correction (q < 0.05). (**B**) Pathway-level analysis of COX-derived metabolites grouped by their shared substrate, arachidonic acid (AA) and eicosapentaenoic acid (EPA). Bars represent the mean log_2_ fold change (TON/Sham), and each dot reflects the average of all COX-derived AA and EPA metabolites in a single mouse. All values were above 0, indicating a trend towards increased metabolite levels in TON eyes, but this was not statistically significant (q < 0.10).

Compared to the LOX and CYP pathways, relatively few COX-derived bioactive lipids were detected. No statistically significant difference was observed in individual COX metabolites (Figure 4A) or in pathway-level comparisons of AA- and EPA-derived subgroups (Figure 4B). However, all metabolites showed log_2_ FC above 0, suggesting a potential upward trend in COX activity following TON. Complete log_2_ FC values for individual metabolites and their pathway-averaged groupings are provided in Supplementary Table S2.

## 4. Discussion

TON remains a clinical challenge due to the lack of effective neuroprotective or regenerative therapies. This study expands on preliminary results presented at the ARVO 2024 meeting [29]. Although PUFA-derived bioactive lipids have been implicated in various neurodegenerative and inflammatory conditions, their role in the context of TON has not been previously explored. This study is the first to target lipidomic profiling of ω−3 and ω−6 PUFA-derived bioactive lipid metabolites in the retina following TON.

In this study, we observed consistent but non-significant trends towards increased expression of bioactive lipid mediator across all three enzymatic pathways—LOX, CYP (including sEH-processed metabolites), and COX. While limited by a small sample size, these early findings suggest potential pathway-specific responses to optic nerve injury that may reflect early lipid signaling dynamics in retinal neuroinflammation or repair. Below, we discuss how select metabolites from each pathway relate to existing literature and potential mechanisms of injury or recovery.

### 4.1. LOX Pathway

In this study, LOX metabolites were grouped by their precursor PUFAs, ω−6 and ω−3, with ω−6 PUFAs further categorized into AA and LA metabolites due to their varying effects on inflammation. LOX-derived metabolites, particularly those of AA and LA, showed a consistent trend of increased expression following TON, although without statistical significance. AA-derived 12/15-LOX and 5-LOX metabolite HETEs such as 5-, 12-, and 15-HETEs have been associated with pro-inflammatory signaling and pathological neovascularization in the retina, including in models of diabetic retinopathy [21] and oxygen-induced retinopathy [30,31]. Pro-inflammatory signaling from 5-, 12-, and 15-HETEs also contribute to neuroinflammation and neuronal damage in the context of TBI [20,32]. 12/15-LOX has been implicated in retinal ganglion cell degeneration, and its genetic deletion confers neuroprotection following optic nerve crush injury [33], suggesting that targeting this enzyme may be a therapeutic strategy for TON as well. Our prior studies also support the pro-inflammatory effects of 12-HETE in retinal Müller cells and vasculature [22], further supporting future studies aimed at understanding the role of LOX enzymes in TON’s inflammatory pathway.

In contrast, LA-derived metabolites such as 9- and 13-hydroxyoctadecadienoic acid (HODEs), and their dehydrogenase forms, 9- and 13-oxo-octadecadienoic acid (OxoODE) may exert anti-inflammatory effects via peroxisome proliferator-activated receptor-γ (PPAR-γ) activation [34,35,36]. These lipids have been detected in endothelial and immune cells, and their upregulation in TON retinas may reflect a compensatory anti-inflammatory response. However, the specific retinal cell types producing these lipids and their receptors remain to be identified.

DHA- and EPA-derived LOX metabolites, including hydroxydocosahexaenoic acid (HDoHE) and 12-hydroxyeicosapentaenoic acid (HEPE), also showed upward trends. While these ω−3-derived HdoHEs [35,36,37,38,39] and EPA-derived 12-HEPE [40,41,42,43] have been linked to neuroprotective and anti-inflammatory effects in various studies, their role in the retina following TON remains largely unexplored.

### 4.2. CYP and sEH Pathways

CYP-derived lipids showed a broader range of FCs, with some sEH-processed metabolites—particularly dihydroxy-eicosatrienoic acids (DiHETrEs) and dihydroxy-9Z-octadecenoic acids (DiHOMEs)—demonstrating the most consistent increases. For example, 11,12-EpETrE is further degraded into 11,12-DiHETrE by the sEH enzyme. EpETrEs have been shown to have protective effects such as modulation of blood pressure, modulation of inflammatory cascades, angiogenesis, neurohormone release, and cell proliferation [44]. Upregulation of sEH in TON may result in increased metabolism of EpETrEs into less active DiHETrEs, thereby decreasing the protective effects of EpETrEs. Hence, the observed elevation of DiHETrEs and DiHOMEs (Figure 3) may therefore reflect increased sEH activity, potentially reducing the beneficial effects of upstream EpETrEs.

LA- and DHA-derived CYP metabolites such as epoxyoctadecamonoenoic acids (EpOMEs), DiHOMEs, and epoxydocosapentaenoic acids (EpDPEs) were also detected but showed less consistent trends. While some have been linked to pain modulation or endothelial responses [45,46,47], their relevance to retinal injury remains unclear and merits further investigation.

### 4.3. COX Pathway

Fewer COX-derived metabolites were detected compared to LOX and CYP pathways, with only AA- and EPA-derived prostaglandins reaching a sufficient detection threshold. Although trends were observed in prostaglandin F2α and D-series prostaglandins, no statistically significant differences were seen. Among detected AA-derived metabolites, D12-PGJ2 has shown anti-inflammatory activity by binding to PPAR**γ** and/or working in the NF-ϰB and extracellular signal-regulated kinase signaling pathways [48] unlike other prostaglandins that are primarily involved in inflammatory pathways. Notably, EPA-derived PGD3 and PGJ3 were elevated in TON eyes, which have both been reported to have anti-inflammatory [49] or vasodilatory roles [50,51]. However, in our current study, conclusions on potential COX-mediated ω−3 response are limited by sample size.

Given that corticosteroids are the only currently available pharmacological treatment option for TON, this study’s observations highlight the importance of targeting LOX, CYP, and sEH enzymes to expand on possible treatment methods. However, this pilot study is limited by its small sample size, which likely reduced power to detect significant differences, especially at the individual metabolite level. Moreover, analysis was restricted to the retina, and did not include optic nerve tissue, which is the primary site of TON injury. Future studies will expand lipidomic profiling to the optic nerve and investigate phospholipase A2 activity—upstream of LOX enzyme. Additionally, the variability observed across animals suggests that biological or technical replicates may further clarify which lipid changes are robust features of TON. Lastly, in discussing our results, it is also important to note that many of these lipid mediators have dual or context-dependent roles depending on the cellular microenvironment, receptor availability, and disease context, which presents a challenge when attempting to translate findings into clinical strategies.

In summary, our study showed a consistent trend towards increased retinal levels of PUFA-derived metabolites that is worth further investigation in the future. With studies suggesting that the compartment-syndromic nature of TON causes ischemic effects [15,52], exploring the relationship between bioactive lipid metabolites in the retinal blood supply and identifying their specific receptors in retina and optic neuronal cells will better elucidate the role of these lipids in TON.

## 5. Conclusions

Multiple enzymes are involved in metabolizing PUFAs into bioactive lipid metabolites that play various roles in cellular function and interaction. With increasing interest in bioactive lipids from PUFAs in disease processes involving inflammatory pathways, understanding biomarkers in the retina caused by TON can help determine possible therapeutic targets that are less invasive compared to currently used treatment options. Our data demonstrate that TON is associated with increased levels of several PUFA metabolites. Although these increases did not reach statistical significance, the consistent increasing trend underscores the biological relevance of these pathways and highlights the need for further investigation. These findings are important, as even delicate shifts in the balance between pro- and anti-inflammatory metabolites may critically influence retinal functional outcomes after TON. Future studies are warranted to precisely define this balance and to explore targetable therapeutic strategies.

## Data Availability

The original contributions presented in this study are included in the article/Supplementary Materials. Further inquiries can be directed to the corresponding author.

## Supplementary Materials

The following supporting information can be downloaded at: https://www.mdpi.com/article/10.3390/biom15101450/s1, Figure S1: Overview of lipoxygenase (LOX), cyclooxygenase (COX), and cytochrome-450 (CYP) metabolites of ω−6 and ω−3 PUFAs. ω−6 PUFAs are shown in orange boxes (linoleic and arachidonic acids) and ω−3PUFAs are shown in green boxes (eicosapentaenoic and docosahexaenoic acids). Soluble epoxide hydrolase (sEH) further metabolizes some of the bioactive lipids.; Table S1: Multiple reaction monitoring (MRM) transitions used in fatty acyl lipidomic analysis; Table S2: Per-mouse log_2_ fold change (FC) values for all bioactive lipid metabolites detected in the retina following traumatic optic neuropathy (TON), grouped by metabolizing enzyme [lipoxygenase (LOX), cytochrome P450 (CYP)] and polyunsaturated fatty acid (PUFA) substrate [arachidonic acid (AA), linoleic acid (LA), eicosapentaenoic acid (EPA), and docosahexaenoic acid (DHA)]. Pathway-level log_2_ fold change averages were calculated for each mouse by averaging across all metabolites derived from a given enzyme–PUFA combination. These pathway-level values were used to generate both the individual-mouse heatmap (Figure 1) and the grouped pathway bar graphs in Figure 2B, Figure 3B and Figure 4B.

## Abbreviations

The following abbreviations are used in this manuscript:

TON	Traumatic optic neuropathy
RGC	Retinal ganglion cell
ERG	Electroretinogram
BRN3A	Brain-specific homeobox/POU domain protein 3A
Iba1	Ionized calcium-binding adapter molecule 1
TBI	Traumatic brain injury
PUFA	Polyunsaturated fatty acid
AA	Arachidonic acid
LOX	Lipoxygenase
HETE	Hydroxyeicosatetraenoic acid
CYP	Cytochrome P450
COX	Cyclooxygenase
COI	Controlled orbital impact
ARVO	Association for Research in Vision and Ophthalmology
LA	Linoleic acid
DHA	Docosahexaenoic acid
EPA	Eicosapentaenoic acid
FC	Fold change
EpOME	Epoxyoctadecamonoenoic acid
EpETrE	Epoxyeicosatrienoic acid
DiHOME	Dihydroxy-9Z-octadecenoic acid
DiHETrE	Dihydroxy-eicosatrienoic acid
sEH	Soluble epoxide hydrolase
HODE	Hydroxyoctadecadienoic acid
OxoODE	Oxoctadecadienoic acid
PPAR-γ	Peroxisome proliferator-activated receptor-γ
HDoHE	Hydroxydocosahexaenoic acid
HEPE	Hydroxyeicosapentaenoic acid
NF-ϰB	Nuclear factor-ϰB
EpDPE	Epoxydocosapentaenoic acid
DiHETE	Dihydroxy-5Z,8Z,11Z,17Z-eicosatetraenoic acid
PG	Prostaglandin
