# Supplementary material for: Alterations of Bioactive Lipid Profiles in the Retina Following Traumatic Optic Neuropathy in Mice
Source: Biomolecules. 2025 Oct 14;15(10):1450. doi: 10.3390/biom15101450 (PMC12564875; doi:10.3390/biom15101450)
Supplement: Supplementary file 1 [file biomolecules-15-01450-s001.zip › Supplementary Tables.pdf]

# Supplementary Materials

## Supplementary Table S1.

| Component Name | IS Name | Expected RT (min) | Precursor (m/z) | Fragment (m/z) |
|---|---|---:|---:|---:|
| PGE1-D4 | N/A | 6.69 | 357.2 | 277.1 |
| RvD2-D5 | N/A | 6.92 | 380.3 | 175.1 |
| LTB4-D4 | N/A | 10.22 | 339.2 | 197.2 |
| 15-HETE-D8 | N/A | 12.58 | 327.2 | 226.1 |
| 14,15-EpETrE-D11 | N/A | 13.34 | 330.3 | 219.1 |
| 13,14-dh-PGE1 | PGE1-D4 | 7.19 | 355.2 | 237.1 |
| 13,14-dh-15k-PGE1 | PGE1-D4 | 7.22 | 353.2 | 237.1 |
| D17-PGE1 | PGE1-D4 | 8.48 | 351.2 | 271.1 |
| PGE1 | PGE1-D4 | 6.95 | 353.2 | 273.2 |
| 15(R)-PGE1 | PGE1-D4 | 6.4 | 353.2 | 273.1 |
| 15-keto PGE1 | PGE1-D4 | 6.3 | 351.2 | 237.1 |
| Bicyclo PGE1 | PGE1-D4 | 8.7 | 335.2 | 209.1 |
| 19(R)-hydroxy PGE1 | PGE1-D4 | 2.83 | 369.2 | 289.1 |
| 2,3-dinor PGE1 | PGE1-D4 | 5.3 | 325.2 | 245.1 |
| PGE2 | PGE1-D4 | 6.43 | 351.2 | 271.1 |
| 15-keto PGE2 | PGE1-D4 | 6.3 | 349.2 | 235.1 |
| 13,14-dh-15k-PGE2 | PGE1-D4 | 6.7 | 351.2 | 235.1 |
| Bicyclo PGE2 | PGE1-D4 | 8.19 | 333.2 | 175.1 |
| PGA2 | PGE1-D4 | 8.3 | 333.2 | 271.1 |
| 19(R)-OH PGE2 & 20-OH PGE2 | PGE1-D4 | 2.96 | 367.2 | 287.1 |
| tetranor PGEM | PGE1-D4 | 2.1 | 327.2 | 291.1 |
| PGE3 | PGE1-D4 | 2.19 | 349.2 | 269.1 |
| PGD2 | PGE1-D4 | 6.85 | 351.2 | 271.1 |
| PGJ2 | PGE1-D4 | 8.2 | 333.2 | 189.1 |
| D12-PGJ2 | PGE1-D4 | 6.7 | 333.2 | 189.1 |
| 15d-D12,14-PGJ2 | PGE1-D4 | 11.55 | 315.2 | 203.1 |
| 13,14-dh-15k-PGD2 | PGE1-D4 | 7.8 | 351.2 | 207.1 |
| PGD3 | PGE1-D4 | 6.22 | 349.2 | 269.1 |
| 15d-D12,14-PGJ3 | PGE1-D4 | 10.1 | 313.2 | 201.1 |
| PGF1a | PGE1-D4 | 7 | 355.2 | 211.1 |
| PGF2a | PGE1-D4 | 7.25 | 353.2 | 193.1 |
| 15-keto PGF2a | PGE1-D4 | 6.5 | 351.2 | 217.1 |
| 13,14-dh-15k-PGF2a | PGE1-D4 | 7.7 | 353.2 | 195.1 |
| 19(R)-OH PGF2a & 20-OH PGF2a | PGE1-D4 | 2.95 | 369.2 | 193.1 |
| PGF3a | PGE1-D4 | 6.1 | 351.2 | 193.1 |

| Component Name | IS Name | Expected RT (min) | Precursor (m/z) | Fragment (m/z) |
|---|---|---|---|---|
| 8-isoPGF2a & 11bPGF2a | PGE1-D4 | 6.2 | 353.2 | 193.1 |
| iPF-VI | PGE1-D4 | 7.02 | 353.2 | 115.1 |
| 6-keto PGF1a | PGE1-D4 | 4.74 | 369.2 | 163.1 |
| 6-keto PGE1 | PGE1-D4 | 4.3 | 367.2 | 143.1 |
| 6,15-diketo PGFa | PGE1-D4 | 4.79 | 369.2 | 267.1 |
| TXB2 | PGE1-D4 | 6.35 | 369.2 | 195.1 |
| 11-dh-TXB2 | PGE1-D4 | 6.8 | 367.2 | 161.1 |
| 2,3-dinor TXB2 | PGE1-D4 | 5.01 | 341.2 | 167.1 |
| 11-dh-2,3-dinor TXB2 | PGE1-D4 | 5.1 | 339.2 | 197.1 |
| TXB3 | PGE1-D4 | 5.2 | 367.2 | 195.1 |
| 11-dh TXB3 | PGE1-D4 | 5.4 | 365.2 | 161.1 |
| LTB4 | LTB4-D4 | 10.41 | 335.2 | 195.1 |
| 12-OxoLTB4 | LTB4-D4 | 10.3 | 333.2 | 179.1 |
| 20-hydroxy LTB4 | LTB4-D4 | 5.21 | 351.2 | 195.1 |
| 20-COOH LTB4 | LTB4-D4 | 2.75 | 365.2 | 195.1 |
| 18-carboxy dinor LTB4 | LTB4-D4 | 2.4 | 337.2 | 195.1 |
| LTB5 | LTB4-D4 | 9.7 | 333.2 | 195.1 |
| 5(S),6(S)-DiHETE | LTB4-D4 | 12.5 | 335.2 | 163.1 |
| 5(S),12(S)-DiHETE | LTB4-D4 | 11.67 | 335.2 | 195.2 |
| 5(S),15(S)-DiHETE | LTB4-D4 | 10.05 | 335.2 | 201.1 |
| 8(S),15(S)-DiHETE | LTB4-D4 | 9.45 | 335.2 | 235.1 |
| 5(S),15(S)-DiHEPE | LTB4-D4 | 10.3 | 333.2 | 253.2 |
| 9-HODE | 15-HETE-D8 | 13 | 295.2 | 171.1 |
| 13-HODE | 15-HETE-D8 | 12.55 | 295.2 | 195.1 |
| 9-HOTrE | 15-HETE-D8 | 11.55 | 293.2 | 171.1 |
| 13-HOTrE | 15-HETE-D8 | 11.65 | 293.2 | 195.1 |
| 13-HOTrE(g) | 15-HETE-D8 | 11.83 | 293.2 | 193.1 |
| 11-HEDE | 15-HETE-D8 | 14.79 | 323.2 | 199.1 |
| 15-HEDE | 15-HETE-D8 | 14.75 | 323.2 | 223.1 |
| 8-HETrE | 15-HETE-D8 | 14.16 | 321.2 | 157.1 |
| 5-HETrE | 15-HETE-D8 | 16 | 321.2 | 115.1 |
| 5-HETE | 15-HETE-D8 | 14.44 | 319.2 | 115.1 |
| 8-HETE | 15-HETE-D8 | 13.65 | 319.2 | 155.1 |
| 9-HETE | 15-HETE-D8 | 13.92 | 319.2 | 151.1 |
| 11-HETE | 15-HETE-D8 | 13.25 | 319.2 | 167.1 |
| 12-HETE | 15-HETE-D8 | 13.53 | 319.2 | 179.1 |
| 15-HETE | 15-HETE-D8 | 13.06 | 319.2 | 219.2 |
| 20-HETE | 15-HETE-D8 | 12.27 | 319.2 | 257.1 |
| tetranor 12-HETE | 15-HETE-D8 | 10.35 | 265.2 | 109.1 |
| 12-HHTrE | 15-HETE-D8 | 10.59 | 279.2 | 163.1 |

| Component Name | IS Name | Expected RT (min) | Precursor (m/z) | Fragment (m/z) |
|---|---|---|---|---|
| 5-HEPE | 15-HETE-D8 | 13 | 317.2 | 115.1 |
| 8-HEPE | 15-HETE-D8 | 12.2 | 317.2 | 155.1 |
| 9-HEPE | 15-HETE-D8 | 12.45 | 317.2 | 149.1 |
| 11-HEPE | 15-HETE-D8 | 11.95 | 317.2 | 167.1 |
| 12-HEPE | 15-HETE-D8 | 12.2 | 317.2 | 179.1 |
| 15-HEPE | 15-HETE-D8 | 13.1 | 317.2 | 219.1 |
| 18-HEPE | 15-HETE-D8 | 11.67 | 317.2 | 259.1 |
| 4-HDoHE | 15-HETE-D8 | 14.8 | 343.2 | 101.1 |
| 7-HDoHE | 15-HETE-D8 | 13.91 | 343.2 | 141.1 |
| 8-HDoHE | 15-HETE-D8 | 14.03 | 343.2 | 189.1 |
| 10-HDoHE | 15-HETE-D8 | 13.51 | 343.2 | 153.1 |
| 11-HDoHE | 15-HETE-D8 | 13.65 | 343.2 | 121.1 |
| 13-HDoHE | 15-HETE-D8 | 13.24 | 343.2 | 193.1 |
| 14-HDoHE | 15-HETE-D8 | 13.34 | 343.2 | 161.1 |
| 16-HDoHE | 15-HETE-D8 | 13 | 343.2 | 233.1 |
| 17-HDoHE | 15-HETE-D8 | 13.08 | 343.2 | 201.1 |
| 20-HDoHE | 15-HETE-D8 | 12.78 | 343.2 | 241.1 |
| 9(10)-EpOME | 14,15-EpETrE-D11 | 13.76 | 295.2 | 171.1 |
| 12(13)-EpOME | 14,15-EpETrE-D11 | 13.59 | 295.2 | 195.1 |
| 5(6)-EpETrE | 14,15-EpETrE-D11 | 15 | 319.2 | 191.1 |
| 8(9)-EpETrE | 14,15-EpETrE-D11 | 14.67 | 319.2 | 155.1 |
| 11(12)-EpETrE | 14,15-EpETrE-D11 | 14.34 | 319.2 | 167.1 |
| 14(15)-EpETrE | 14,15-EpETrE-D11 | 13.81 | 319.2 | 219.1 |
| 8(9)-EpETE | 14,15-EpETrE-D11 | 13.21 | 317.2 | 155.1 |
| 11(12)-EpETE | 14,15-EpETrE-D11 | 13.1 | 317.2 | 167.1 |
| 14(15)-EpETE | 14,15-EpETrE-D11 | 13.4 | 317.2 | 219.1 |
| 17(18)-EpETE | 14,15-EpETrE-D11 | 12.41 | 317.2 | 259.1 |
| 7(8)-EpDPE | 14,15-EpETrE-D11 | 14.9 | 343.2 | 141.1 |
| 10(11)-EpDPE | 14,15-EpETrE-D11 | 14.5 | 343.2 | 153.1 |
| 13(14)-EpDPE | 14,15-EpETrE-D11 | 14.4 | 343.2 | 193.1 |
| 16(17)-EpDPE | 14,15-EpETrE-D11 | 14.2 | 343.2 | 233.1 |
| 19(20)-EpDPE | 14,15-EpETrE-D11 | 13.7 | 343.2 | 241.1 |
| 9,10-DiHOME | LTB4-D4 | 10.72 | 313.2 | 201.1 |
| 12,13-DiHOME | LTB4-D4 | 10.34 | 313.2 | 183.1 |
| 5,6-DiHETrE | LTB4-D4 | 13.62 | 337.2 | 145.1 |
| 8,9-DiHETrE | LTB4-D4 | 12.5 | 337.2 | 185.1 |
| 11,12-DiHETrE | LTB4-D4 | 11.78 | 337.2 | 167.1 |
| 14,15-DiHETrE | LTB4-D4 | 11.25 | 337.2 | 207.1 |
| 5,6-DiHETE(EPA) | LTB4-D4 | 11.1 | 335.2 | 145.1 |
| 19,20-DiHDoPE | LTB4-D4 | 11.08 | 361.2 | 273.2 |

| Component Name | IS Name | Expected RT (min) | Precursor (m/z) | Fragment (m/z) |
|---|---|---|---|---|
| 9-OxoODE | 15-HETE-D8 | 12.45 | 293.2 | 185.1 |
| 13-OxoODE | 15-HETE-D8 | 12.09 | 293.2 | 113.1 |
| 9-OxoOTrE | 15-HETE-D8 | 11.12 | 291.2 | 185.1 |
| 15-OxoEDE | 15-HETE-D8 | 14.53 | 321.2 | 223.1 |
| 5-oxoETE | 15-HETE-D8 | 14.1 | 317.2 | 203.1 |
| 12-OxoETE | 15-HETE-D8 | 13.1 | 317.2 | 153.1 |
| 15-OxoETE | 15-HETE-D8 | 12.45 | 317.2 | 219.1 |
| LXA4 | LTB4-D4 | 8.12 | 351.2 | 217.1 |
| 15-epi LXA4 | LTB4-D4 | 9.4 | 351.3 | 217.2 |
| 15-oxo LXA4 | LTB4-D4 | 8.3 | 349.2 | 189 |
| LXA5 | LTB4-D4 | 6.94 | 349.2 | 215.1 |
| LXB4 | LTB4-D4 | 8.01 | 351.2 | 221.1 |
| RvD1 & AT-RvD1 | RvD2-D5 | 8.05 | 375.2 | 141.1 |
| RvD2 | RvD2-D5 | 7.4 | 375.2 | 175.1 |
| RvD3 | RvD2-D5 | 7.6 | 375.3 | 147.1 |
| AT-RvD3 | RvD2-D5 | 7.1 | 375.2 | 147.2 |
| RvD4 | RvD2-D5 | 9.39 | 375.3 | 131.1 |
| AT-RvD4 | RvD2-D5 | 9.3 | 375.2 | 131.1 |
| 8-oxoRvD1 | RvD2-D5 | 7.8 | 373.2 | 261.1 |
| 17-oxoRvD1 | RvD2-D5 | 11.45 | 373.2 | 231.1 |
| 22-OH-PD1 | RvD2-D5 | 6.1 | 375.2 | 204.2 |
| RvE1 | RvD2-D5 | 4.9 | 349.2 | 195.1 |
| RvD5 | LTB4-D4 | 10.22 | 359.2 | 199.1 |
| RvD6 (4,17-DiHDoHE) | LTB4-D4 | 10.5 | 359.2 | 159.1 |
| AT-RvD6 (4_17-DiHDoHE) | LTB4-D4 | 10.9 | 359.2 | 215.1 |
| RvD5(n-3,DPA) (7,17-DiHDoPE) | LTB4-D4 | 10.3 | 361.2 | 219.1 |
| AT-RvD5(n-3_DPA) (7_17-DiHDoPE) | LTB4-D4 | 10.4 | 361.2 | 201.1 |
| RvE2 | LTB4-D4 | 9.1 | 333.2 | 213.1 |
| RvE3 | LTB4-D4 | 10.7 | 333.2 | 201.1 |
| PD1 | LTB4-D4 | 11.16 | 359.2 | 206.1 |
| AT-PD1 | LTB4-D4 | 10.1 | 359.2 | 206.2 |
| PDx (10S,17S-DiHDoHE) | LTB4-D4 | 9.7 | 359.2 | 153.1 |
| PD1(n-3,DPA) | LTB4-D4 | 10.3 | 361.2 | 155.1 |
| Maresin1 | LTB4-D4 | 10.23 | 359.2 | 177.1 |
| Maresin2 | LTB4-D4 | 10.63 | 359.2 | 221.2 |
| MaR1(n-3,DPA) | LTB4-D4 | 10.6 | 361.2 | 223.1 |

**Supplementary Table S2.**

| Metabolizing Enzyme | Polyunsaturated Fatty Acid (PUFA) | Bioactive Lipid Metabolite | Mouse 1 $Log_2$ FC | Mouse 2 $Log_2$ FC | Mouse 3 $Log_2$ FC | Mouse 1 $Log_2$ FC (Pathway Avg) | Mouse 2 $Log_2$ FC (Pathway Avg) | Mouse 3 $Log_2$ FC (Pathway Avg) |
|---|---|---|---|---|---|---|---|---|
| Lipoxygenase (LOX) | AA | 12-HETE | 1.565 | 0.184 | 0.116 | 1.358 | -0.271 | 0.256 |
| | | 15-HETE | 1.384 | -0.387 | 0.359 | | | |
| | | 5-HETE | 0.864 | 0.383 | 0.522 | | | |
| | | 11-HETE | 1.557 | 0.017 | 0.283 | | | |
| | | 8-HETE | 1.221 | 0.211 | 0.410 | | | |
| | | 9-HETE | 1.129 | -0.393 | 0.316 | | | |
| | | tetranor-12-ETE | 1.785 | -1.908 | -0.215 | | | |
| | LA | 13-OxoODE | 2.770 | 0.168 | -0.044 | 2.758 | 0.144 | 0.238 |
| | | 9-OxoODE | 2.472 | 0.143 | 1.099 | | | |
| | | 13-HODE | 2.787 | 0.154 | -0.201 | | | |
| | | 9-HODE | 3.004 | 0.109 | 0.098 | | | |
| | EPA | 12-HEPE | 1.394 | 0.059 | 0.169 | 1.394 | 0.059 | 0.169 |
| | DHA | 14-HDoHE | 1.182 | 0.087 | 0.373 | 1.192 | 0.167 | 0.038 |
| | | 4-HDoHE | 1.209 | 0.093 | 0.252 | | | |
| | | 8-HDoHE | 1.053 | 1.100 | -1.161 | | | |
| | | 16-HDoHE | 1.211 | -0.124 | 0.557 | | | |
| | | 11-HDoHE | 1.209 | -0.066 | 0.049 | | | |
| | | 13-HDoHE | 1.356 | -0.057 | 0.638 | | | |
| | | 10-HDoHE | 1.211 | -0.014 | -0.037 | | | |
| | | 7-HDoHE | 1.110 | 0.315 | -0.364 | | | |
| Cytochrome P450 (CYP) | AA | 5,6-EpETrE | 0.337 | 0.944 | -1.703 | 1.257 | 0.524 | -0.121 |
| | | 14,15-DiHETrE | 1.709 | 0.434 | 0.302 | | | |
| | | 11,12-DiHETrE | 1.188 | 0.073 | 1.364 | | | |
| | | 20-HETE | 1.473 | -0.034 | -0.689 | | | |
| | | 11,12-EpETrE | 1.580 | 1.201 | 0.119 | | | |
| | LA | 9(10)-EpOME | 2.149 | -0.784 | -0.151 | 2.460 | -0.350 | 0.367 |

| | | | | | | | | |
|---|---|---|---|---|---|---|---|---|
| | | 12(13)-EpOME | 2.856 | -0.488 | -1.002 | | | |
| | | 9,10-DiHOME | 2.280 | 0.329 | 0.983 | | | |
| | | 12(13)-DiHOME | 2.554 | -0.458 | 1.638 | | | |
| | EPA | 5(S),6(S)-DiHETE | 3.068 | 0.977 | | 3.068 | 0.977 | |
| | DHA | 20-HDoHE | 1.314 | 0.375 | 0.157 | 1.849 | -0.056 | 0.217 |
| | | 19(20)-EpDPE | 1.858 | 0.144 | 0.499 | | | |
| | | 10(11)-EpDPE | 1.895 | 0.340 | -0.131 | | | |
| | | 13(14)-EpDPE | 2.109 | -0.619 | 0.343 | | | |
| | | 16(17)-EpDPE | 2.068 | -0.520 | | | | |
| | sEH | 14,15-DiHETrE | 1.709 | 0.434 | 0.302 | 1.933 | 0.095 | 1.072 |
| | | 11,12-DiHETrE | 1.188 | 0.073 | 1.364 | | | |
| | | 9,10-DiHOME | 2.280 | 0.329 | 0.983 | | | |
| | | 12(13)-DiHOME | 2.554 | -0.458 | 1.638 | | | |
| Cyclooxygenase (COX) | AA | PGE2 | 1.167 | -0.075 | -0.105 | 1.144 | 0.181 | 0.599 |
| | | TXB2 | 2.298 | -0.889 | 0.890 | | | |
| | | PGF2a | 1.927 | 0.234 | 0.566 | | | |
| | | D12-PGJ2 | -2.270 | 2.165 | | | | |
| | | PGD2 | 2.224 | -0.383 | 1.046 | | | |
| | | 15-keto PGE2 | 1.519 | 0.033 | | | | |
| | EPA | PGD3 | 3.037 | 0.111 | | 2.534 | 0.268 | 0.143 |
| | | 15d-D12,14-PGJ3 | 2.030 | 0.426 | 0.143 | | | |
