# Supplementary figures and images for: Alterations of Bioactive Lipid Profiles in the Retina Following Traumatic Optic Neuropathy in Mice
Source: Biomolecules. 2025 Oct 14;15(10):1450. doi: 10.3390/biom15101450 (PMC12564875; doi:10.3390/biom15101450)

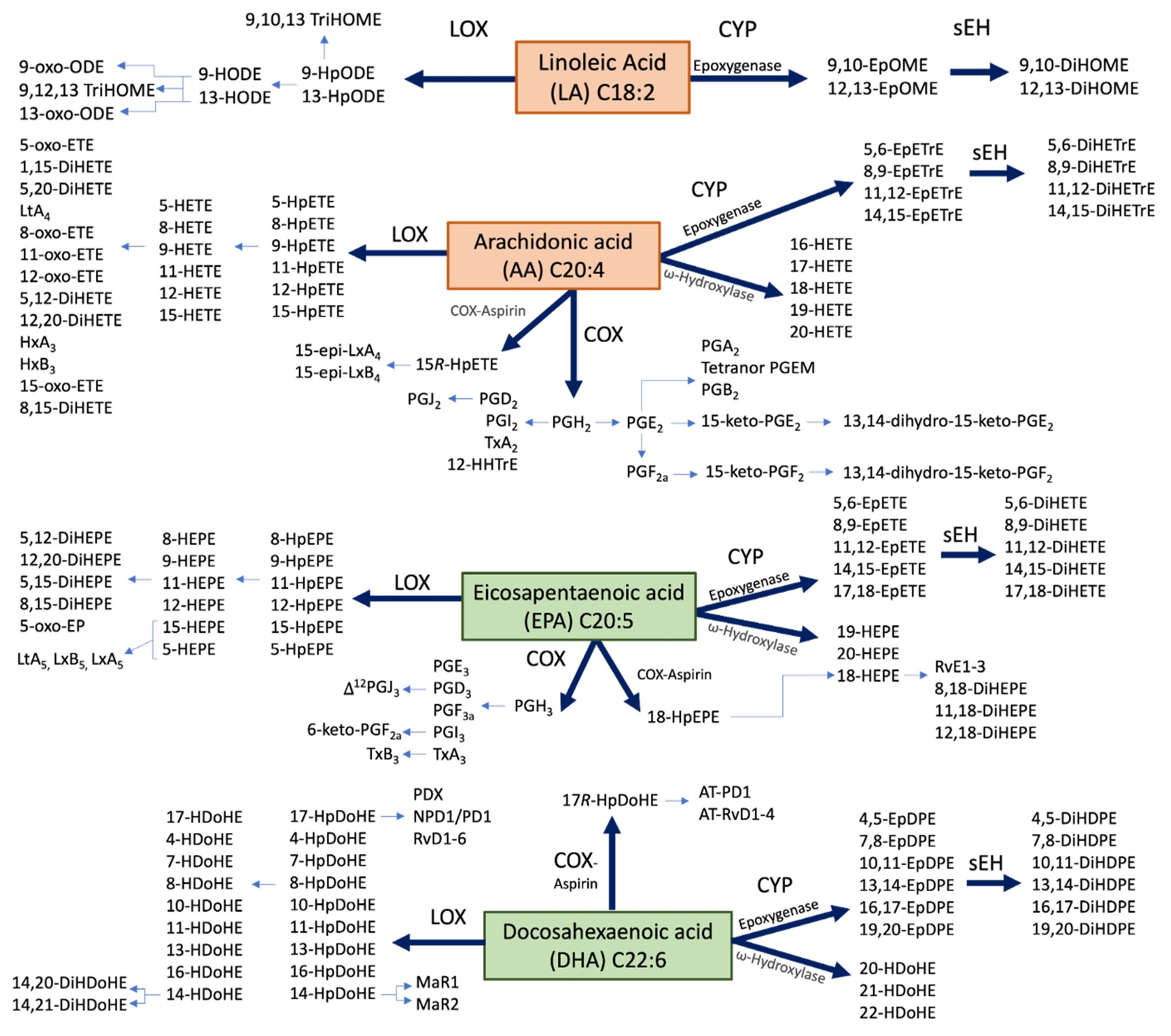

Supplement: Supplementary file 1 [file biomolecules-15-01450-s001.zip › Supplementary Figure 1.jpg]
